# Strengthening the evidence to improve health outcomes of children with perinatal HIV exposure

**DOI:** 10.1002/jia2.26160

**Published:** 2023-11-01

**Authors:** Sonia Lee, Susannah Allison, Pim Brouwers

**Affiliations:** ^1^ Eunice Kennedy Shriver National Institute of Child Health and Human Development National Institutes of Health Bethesda Maryland USA; ^2^ National Institute of Mental Health National Institutes of Health Bethesda Maryland USA

**Keywords:** adolescents, children, epidemiology, HEU, HIV‐exposed uninfected, perinatal

## Abstract

**Introduction:**

The number of children exposed to HIV and possibly to antiretroviral therapy (ART) in utero and during breastfeeding and are uninfected (HEU) globally will continue to increase from the estimated 15.9 million in 2021.

**Discussion:**

There are still significant gaps in our understanding of the impact of HIV and/or ART exposure in children who are HEU, in terms of prevalence/incidence and severity on health and wellbeing, and long after exposure has ended. While there have been substantial programmatic efforts to support the elimination of vertical transmission of HIV, additional rigorous research is needed to better understand the biological, (psycho)social and structural factors contributing to optimal health for populations who are HEU. Furthermore, the best approaches to address and study the gaps in understanding also need to be explored. Given the scope of the problem including the large numbers of affected people as well as the often limited and competing in‐country resources for populations affected by HIV, novel methodologies, including multi‐level approaches and advanced analytics, need to be considered.

**Conclusions:**

A growing population of children who are HEU are maturing into adolescence and young adulthood. Research to advance understanding of the possible negative long‐term effects of HIV and/or ART exposure in these youth is supported by the US National Institutes of Health. Both large epidemiological studies and smaller more comprehensive cohort studies may be required to address the complexity of the issue. Integrating both types of studies could allow the establishment of more reliable and validated predictions of which youth who are HEU are at the highest risk for specific negative health outcomes, such as mental health and neurocognitive disorders, and which interventional approaches may be most successful to address specific deficits both in terms of prevention and treatment. Finally, these goals can be more rapidly achieved with data science efforts, data harmonisation between studies and with sustainable data‐sharing practices. It is important to expand the commitment to research to identify biological, social and structural drivers, to develop screening tools, and impactful and contextually appropriate interventions to address the health and wellbeing of children who are HEU from birth through adulthood.

## 1 INTRODUCTION

The number of children exposed to HIV and possibly to antiretroviral therapy (ART) in utero and during breastfeeding who are uninfected (HEU) will continue to increase from the estimated 15.9 million children in 2021 [1, 2]. Furthermore, an increasing number of these children are now growing into adolescence (13–18 years of age) and young adulthood (18–21 years of age) [3]. These biological exposures put this population at increased risk for negative health outcomes. A number of studies indicate that children who are HEU are at increased risk for complicated maternal pregnancy and delivery, increased rates of preterm delivery, mortality, infectious morbidity, immune abnormalities and impaired growth [4]. These complications may also act as mediators of the delays in cognitive, motor, language and neurodevelopment that have been reported in some studies [5], mainly focused on younger age groups. In addition to the more biologic factors, mental health and neurocognitive outcomes will also be moderated by psychosocial and behavioural factors in addition to social determinants of health [6]. There may also be a disproportionate impact of structural and health inequalities on the youth growing up in an environment affected by HIV.

A recent systematic review and meta‐analysis highlighted the need for large high‐quality longitudinal studies to assess the neurodevelopmental trajectories of children who are HEU [7]. Furthermore, psychosocial factors and social determinants of health also may play a significant and increasing role over time moderating the possible HIV and ART‐related negative health outcomes of youth who are HEU. For example, inequitable access to healthcare services and education, low socio‐economic status and HIV stigma continue to affect children who are HEU growing up in HIV‐affected environments. Slogrove described a “package of risks,” including potentially adverse factors beyond ART and HIV exposure, such as social and structural inequalities, exposures to other infectious diseases, and maternal morbidity and mortality, that also adversely affect the development of children who are HEU [8].

It is important to understand the underlying mechanisms and compounding risk factors for increased negative health outcomes to predict those at highest risk and inform appropriate targeted management and intervention strategies that optimise the overall wellness of the growing population of youth and young adults who are HEU. As most psychiatric disorders emerge in late childhood and adolescence, these disorders may not yet be apparent; therefore, it is important for older populations who are HEU to be assessed for mental health symptoms and disorders in order for them to be rapidly addressed [9]. Factors that extend beyond in‐utero exposures also must be considered when evaluating the mechanisms or risk factors impacting health outcomes among older populations who are HEU. Given the breadth of both heterogeneous etiologic factors and health outcomes in populations who are HEU, the large affected populations of children, adolescents and young adults, as well as the often limited and competing in‐country resources to address health and other inequities, research with a focus on adolescents and young adults that includes possible targeted interventions for mental and neurocognitive health need to be more highly prioritised.

This commentary focuses on research gaps related to the long‐term effects of in‐utero exposure to HIV and/or ART on mental health and neurocognitive development as populations who are HEU age into adolescence and young adulthood, highlights major US National Institutes of Health (NIH)‐funded research programmes with populations who are HEU and proposes future research priorities and directions.

## 2 DISCUSSION

### 2.1 Selected NIH research programmes with paediatric populations who are HEU

The NIH has a mission to seek fundamental knowledge about the nature and behaviour of living systems and the application of that knowledge to enhance health, lengthen life, and reduce illness and disability. With respect to HIV research priorities, this includes funding a broad research agenda, in alignment with the NIH Strategic Plan for HIV and HIV‐Related Research [10]. Specifically, Strategic Goal 1 to “Advance rigorous and innovative research to end the HIV pandemic and improve the health of people with, at risk for, and affected by HIV across the lifespan” addresses the need for additional research in understanding the impact of ART and HIV exposure and fostering the bridge from research findings to clinical and community policies and practice. Several NIH scientific programmes focus on research with populations who are HEU and provide opportunities to further the field. Innovative approaches, including novel and rigorous study designs, are needed to identify those with the highest need for evidence‐based interventions that would be effective and implementable. Studies to link infant and child health outcomes with maternal health records during pre‐pregnancy, pregnancy and breastfeeding are vital for this research.

Maintaining this information through the transition to adolescent and adult care is also critical but will be difficult. Study populations of individuals who are HEU will need to be large enough to allow for sufficient power to generate new insights around the impact of HIV and ART exposure on mental health and neurodevelopmental outcomes, because of possible complex interactions between individual, maternal, and environmental factors and low prevalence or small effect sises of associated abnormalities. Efforts to encourage and facilitate interactions among separate cohorts need to be strongly promoted to improve the sharing of approaches, methodologies, standardised assessments and data analyses, to develop harmonisation standards, and to establish sustainable data‐sharing plans.

In response to these objectives, in 2020, the *Eunice Kennedy Shriver* National Institute of Child Health and Human Development and the National Institute of Mental Health supported a programme on HIV epidemiology research that establishes, monitors and evaluates large cohorts to understand the effects of in‐utero HIV and/or ART exposure on mortality, morbidity and growth in the United States and/or international settings. Grants were issued to support cohorts of populations who are HEU in Botswana, Kenya, Malawi, South Africa and Zimbabwe [11]. Expected outcomes from the utilisation of innovative epidemiologic approaches to assess health in these cohorts include knowledge that can address care gaps in growth and nutrition, hearing, mental health, cognition, academic achievement and cardiovascular fitness.

Another NIH development that could further facilitate the progress in follow‐up of populations who are HEU in Africa is the NIH Common Fund's Harnessing Data Science for Health Discovery and Innovation in Africa (DS‐I Africa) [12]. This programme leverages data science technologies and prior NIH investments to develop solutions to the continent's most pressing public health problems through a robust ecosystem of new partners from academic, government and private sectors.

In the United States, the NIH has also provided long‐standing research funding support for the Pediatric HIV/AIDS Cohort Study (PHACS), including the Surveillance and Monitoring for ART Toxicities (SMARTT) study to evaluate the long‐term safety of antiretroviral (ARV) medications taken during pregnancy among women living with HIV (WLHIV) and their children born without HIV [13, 14, 15]. The PHACS SMARTT study is the largest comprehensive study of pregnant WLHIV in the United States, and one of the only large studies to follow children born to WLHIV beyond infancy into their adolescence. A major focus of the SMARTT study is the evaluation of effects of maternal exposures to ARVs, prescribed medications and substance use during pregnancy on health outcomes in their infants and children. These health outcomes cross a wide spectrum of domains, including growth, neurodevelopment, cardiometabolic, hearing and language, and neurologic functioning. While there are clearly many differences between this cohort and similar populations who are HEU in low‐ or middle‐income countries (LMICs), these studies may also provide guidance for more targeted and efficient follow‐up to identify sequelae in populations who are HEU in LMICs.

The NIH further supports additional important collaborative efforts with stakeholders of populations who are HEU to increase awareness and prioritise the dissemination of research findings, identify research gaps and encourage future opportunities. Notably, the 8th Workshop on Children and Adolescents with Perinatal HIV Exposure in 2022 presented up‐to‐date evidence of optimal methods to evaluate neurodevelopmental outcomes in HIV high prevalence settings, as well as child neurodevelopmental outcomes following perinatal HIV exposure [11]. The workshop included researchers, clinicians, programme implementers, policymakers, advocates and parents of youth who are HEU in order to be a catalyst for shaping research agendas and health policies to support this population of children and adolescents and young adults to reach their fullest human potential.

## 3 CONCLUSIONS

### 3.1 Future research directions

To achieve optimal healthcare for populations who are HEU, research priorities need to emphasise empirical investigations of the long‐term effects of HIV and ART exposure in children, adolescents and young adults (see Table 1).

**Table 1:** Future priorities and directions

Larger longitudinal epidemiologic studies, including registries and/or cohorts of children, adolescents and young adults who are HEU, to efficiently identify clinically relevant mental health, behavioural and/or neurocognitive outcomes across the lifespan in ways that minimise participant burden Rigorous, innovative study designs and data science methodologies to identify and focus on early identification of those at highest risk of poor outcomes Focused measurement and assessment of smaller cohorts of populations who are HEU in greatest need of interventions Expanded data harmonisation and sharing efforts Increased ability to identify children, adolescents and young adults who are HEU in other longitudinal research to allow for comparative evaluations to those who are not HIV exposed

Longitudinal research with populations who are HEU may require large epidemiologic studies with innovative limited and possibly remote data collection methods and, importantly, the use of medical records with linkages between mother and child. Furthermore, mathematical predictive modelling using these biomedical cohort data, supplemented with structural and social determinants of health data, may also indicate which mental health and behavioural preventive and therapeutic interventions may be most successful to address the specific impacts of HIV and/or ART perinatal exposure. More comprehensive and neuroscientific approaches would include utilizing smaller cohorts to allow for limited but focused assessments. For example, magnetic resonance imaging technology or the use of biomarkers to assess brain development and functioning may be too costly to use in many settings or large‐scale cohorts, but may be feasible in smaller cohorts [16].

Similarly, evidence‐based screening approaches and protocols for developmental outcomes in children who are HEU that identify the need for further evaluation beyond the early life are a promising area for research and programmatic investments. Providing early opportunities for interventions during the early years of life when the children are still in active care and may support children as well as caregivers [17] may be critical as the likelihood for loss to follow‐up when these children reach adolescence or young adulthood is great. For example, the World Health Organisation Nurturing Care Framework for Early Childhood Development highlights the provision of knowledge, skills, time and material resources to give appropriate childcare as essential for the healthy development of children [18]. The development of effective screening tools can lead to the delivery of differentiated services based on need. Differentiated care or differentiated service delivery is defined as client‐centred approaches that simplify and adapt services, in ways that both better serve individuals and enhance efficiency of care delivery within the healthcare system. Differentiated care supports shifting resources to clients who are the most in need, innovating the way care is delivered through tailored services. There is a growing understanding of how to deliver differentiated care to adults living with HIV, but limited knowledge about how these approaches can be used to target the unique needs of the children of adults living with HIV.

There remain significant barriers to long‐term follow‐up studies of children who are HEU, particularly when they become more independent and age into adolescence and young adulthood, and have increasing autonomy to make medical decisions and actively participate in their healthcare. Disclosure of maternal status may not have occurred, limiting adolescent and young adults’ knowledge of their HIV and/or ARV exposure and preventing their inclusion in studies on the long‐term impact of these exposures. In addition, the lack of clinically relevant manifestations of exposures, as well as the desire to not be different from their HIV‐unexposed peers, may decrease the interest in continuing to participate in such research. Other structural and economic factors may also lead to a loss to follow‐up. A scientific concern here is that this loss may not be random and could bias the results. Approaches to address these barriers or correct for them are needed.

Finally, impactful efforts to improve health outcomes for families with children and adolescents who are HEU will encompass the expertise, input, perspectives and voices of researchers, clinicians, programme implementers, policymakers, advocates and community, and of the children and their families themselves. As we expand efforts to achieve global elimination of vertical transmission, we should not allow optimal health for children and adolescents who are HEU to be far behind. The acknowledgement and integration of appropriate targets for health indicators and outcomes of children, adolescents and young adults who are HEU need to underlie global research partnerships and inform the global HIV agenda.

## COMPETING INTERESTS

The authors have no competing interests to disclose.

## AUTHORS’ CONTRIBUTIONS

SL conceptualised, drafted and revised the manuscript. SA and PB conceptualised and revised the manuscript.

## DISCLAIMER

The content is solely the responsibility of the authors and does not necessarily reflect the official views of the Department of Health and Human Services or the National Institutes of Health.

## Data Availability

Data sharing not applicable to this article as no datasets were generated or analysed during the current study.
